# Factors associated with self-medication in children and the decomposition of rural-urban disparities in China

**DOI:** 10.1186/s12889-021-12137-1

**Published:** 2021-11-18

**Authors:** Jingjing Ge, Xiaxia Sun, Hongdao Meng, Punam Ghimire Risal, Danping Liu

**Affiliations:** 1 Department of Health Related Behavior and Social Medicine, West China School of Public Health and West China Fourth Hospital, Sichuan University, Chengdu, China; 2 Gansu Provincial Maternity and Child-care Hospital, Lanzhou, China; 3 West China Second University Hospital, Sichuan University/West China Women’s and Children’s Hospital, Chengdu, China; 4 School of Aging Studies, College of Behavioral & Community Sciences, University of South Florida, Tampa, FL 33620 USA

**Keywords:** Self-medication, Children, Rural-urban disparities, China

## Abstract

**Background:**

Self-medication in children is one of the greatest threats to children health in China.

**Objectives:**

The purpose of this study was to examine the potential factors associated with self-medication in children and explore rural-urban disparities.

**Methods:**

A total of 2798 children enrolled in the study. Informed consent was obtained from each primary caregiver following a detail explanation about the purpose of the study. Multivariable logistic regression analysis and Oaxaca–Blinder decomposition analysis were used.

**Results:**

The results showed that 38.2% primary caregivers of rural areas self-medicated their children, compared to 18.7% of those in urban areas. The urban primary caregivers with college or above education were more likely to self-medicate their children, while rural primary caregivers with college or above education were less likely to self-medicate their children. Children having unhealthy eating habits were more likely to have been self-medicated by their primary caregivers in urban and rural areas. Urban primary caregivers who spend more than 10 min from home to the nearest medical institution were more likely to self-medicate their children. In rural areas, children aged 3–6 years old, primary caregivers with monthly household income per capita of 1001–3000 Yuan, and children with chronic diseases are another set of enabling factors which impacted on self-medication. Unhealthy eating habits of children were the largest contributor to the rural-urban self-medication gap.

**Conclusions:**

Children’s factors explained the largest portion of the rural-urban difference in self-medication among children. The evidence presented in this study suggests that public health policies addressing rural-urban differences in children’ s factors could serve as an effective method for reducing rural-urban disparities in self-medication among children.

## Background

The use of medicines by individuals to treat self-recognized illnesses or symptoms is defined as self-medication [1]. Self-medication includes several forms through which individuals themselves or the caregivers (for minors) decide, without medical evaluation, which medication they will use for symptom relief and treatment; it involves sharing drugs with other members of the family and social group, using leftovers from previous prescriptions or disrespecting the medical prescription either by prolonging or interrupting the dosage and the administration period prescribed [2]. Children are often given medicines by their primary caregivers. Self-medicated medicines in children included antibiotics, cough and cold medicines (CCMs), vitamins, supplements, and so on [3–7].

Globally, self-medication in children is wide spread, with self-administering of antibiotics is most common with potential serious consequences. A study in Tetovo reported 86.6% of parents using antibiotics without doctors’ prescription [3]. Regarding the other self-medicated medicines in children, Ylinen et al. revealed that the prevalence of the use of Over The Counter (OTC) medicines (excluding vitamins), vitamins, and complementary and alternative medicines (CAMs) in the preceding 2 days were 17.0, 37.0, and 11.0%, respectively [6]. A study in Germany suggested that 25.2% of caregivers had self-medicated their children or adolescents, vitamin preparations were most frequently used with a weighted user prevalence of 4.7%, followed by CCMs (4.4%) and analgesics (3.7%) [7]. Therefore, the self-medicating behavior among primary caregivers of children warrant additional investigations.

In China, self-medication in children is at a critical trend. Although regulations that categorise drugs as prescription only and OTC in China, they are not adequately enforced [8]. Many prescription-only medicines can be purchased without a prescription, including antibiotics, sedatives, tranquillisers, stimulants, and pain relievers [9–11]. For example, Miao’s research showed that 62.0% of parents in Jiangxi province of China had self-medicated their children with antibiotics [4]. Li et al. reported that 35.1% of the parents had taken antibiotics to treat diarrhea of their children without any prescription [5]. Since 1982, China had defined the family planning as a basic national policy, advocating late childbirth, and fewer births, to dampen population growth. Therefore, China has appeared a large number of single-child families. Despite the relaxation of the one-child policy since 2015, the fertility rate of Chinese parents was still low, especially for women with higher educational level and urban family [12]. In the family of only-child, parents often are excessively anxious or worried about the health of the child. If they have difficulty accessing to general practitioners and hospitals, the parents will immediately give the child medicine without doctors’ prescription when their child exhibits symptoms of illness [13].

Some pediatric medications, such as vitamin supplements, are safe if used correctly, i.e. according to the package insert. Nevertheless, parents may have incorrect knowledge about medications, which can lead to inappropriate medication use, thus cause serious damage to health. For example misuse of antibiotics increases the risk of antibiotic resistance [14], excessive vitamin use may increase the risk of cancer and cardiovascular disease [15, 16], misuse of traditional Chinese medicine may cause liver and kidney damage [17]. Aspirin, for example, should not be used in children under 12 months of age, otherwise, adverse drug reactions may occur - Reye syndrome is a life-threatening adverse reaction to aspirin in young children [18]. The dose of children’s medications is different from adults, such as accidental CCMs that have resulted in serious adverse cardiovascular events, these incidents included several infant deaths [19, 20].

The population of China accounts for one fifth of the world’s population, and more than half of the Chinese population lives in rural areas. Individuals with higher education and higher income are usually experience better health [21], while rural people are generally less educated and have lower income levels. Moreover, most medical institutions are located in cities, so people living in rural areas have less access to medical services [22]. Therefore, primary caregivers in rural areas may be more likely to self-medicate their children. For example, using antibiotics without medical prescription was higher frequency in lower socio-economic areas [23]. Yu et al. found that parents living in villages were significantly associated with self -medication of children with antibiotics in China [4].

The difference between urban and rural areas in family caregiver self-medicated their children may contribute to rural–urban children health disparities in China. It is necessary to examine the potential factors of self-medication among children and assessed the contribution of the observed factors to the rural-urban disparities in self-medication, thus to inform policy guidance and interventions to improve their training on appropriate medicine use that should lead to changes in behavior, and reduce the rural-urban children health disparities. The objectives of this study were: (1) to examine factors associated with self-medication in a sample of children who lived in China; and (2) to quantify the degree to which disparities could theoretically be reduced if rural-urban differences in determinants of self-medication were eliminated, this study was to apply Oaxaca–Blinder decomposition [24] to explore rural-urban disparities in self-medication among children.

## Methods

### Setting and participants

**Fig. 1:**
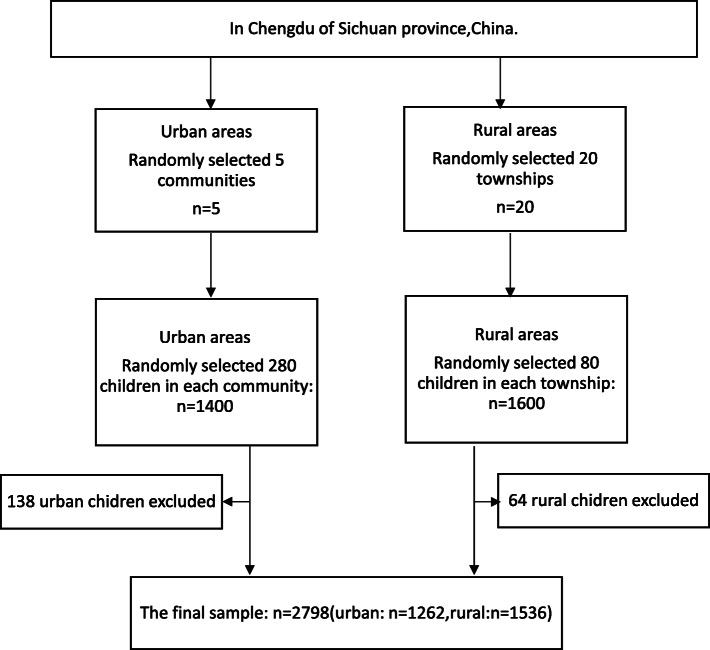
Sampling flow-chat

A cross-sectional survey was conducted in Chengdu, China between February 2016 and June 2016. The sample was selected using two-stage stratified random sampling. In the first stage, we randomly selected 5 communities from urban areas and 20 townships from rural areas in Chengdu of Sichuan Province. In the second stage, 280 children aged 0–6 years were randomly selected from each community, and 80 children aged 0–6 years were randomly selected from each township (Fig. 1). The primary caregivers of children selected were asked to respond anonymously during face-to-face interviews by trained interviewers. Primary caregivers were defined as those who regularly cared for the children, such as parents or grandparents [25]. A total of 2798 participants completed the questionnaires in the end, 1262 participants in urban areas with an effective response rate of 90.1% and 1536 participants in rural areas with an effective response rate of 96.0%. Informed consent was obtained from each primary caregiver following a detail explanation about the purpose of the study.

### Measures

The dependent variable of interest was self-medication of children. Independent variables included 15 measures of characteristics at the children, primary caregivers and household levels.

#### Self-medication in children

Primary caregiver’s self-medication for children was measured by an affirmative response (Yes) to the question: “Did you give your children medicine without the doctor’s permission or advice in prior year?”

#### Children’s characteristics

Children’s characteristics included socio-demographic characteristics, health-related behaviors and health status. The socio-demographic characteristics included gender, age (0–2 and 3–6 years), having medical insurance (yes, no). Health-related behaviors and health status included: having unhealthy eating habits, having chronic diseases, being sick in the 2 weeks prior to the survey, and hospitalized in prior year. The response was “yes” or “no”. In this study, unhealthy eating habits were defined as eating habits that adversely affect children’s health formed in their daily life, such as picky food, partial food, eating more snacks, gluttony, playing or watching TV while eating.

#### Primary caregivers’ characteristics

Primary caregivers’ characteristics included gender, age (<=29, 30–44, 45–59 and > =60 years), education (primary school or below, secondary or high school, college or above), employment (unemployment, nonmanual work, manual work and retirement), primary caregivers often acquire health knowledge (yes or no).

#### Household-level factors

Household factors included monthly household income per capita (<=1000, 1001–3000, and > 3000 Yuan), and walking time from home to the nearest medical institution (<=10 min, > 10 min).

### Statistical analysis

Categorical variables were described with frequencies and percentages by rural and urban areas. Chi-square tests were used to evaluate significant differences with respect to categorical variables. We used multivariable logistic regression analysis to examine factors associated with primary caregiver’s self-medication of children. To investigate the relative contribution of each of the considered factors, we conducted an Oaxaca–Blinder decomposition analysis (OBDA) [24]. This is commonly used to analyze differences in continuous dependent variables, but it can be applied to analyze absolute differences in a binary variable such as self-medication status [26]. OBDA partitions the gap into an “explained” portion and an “unexplained” portion. In this study, the “explained” portion represents the amount by which the rural–urban difference in self-medication among children would be reduced in the hypothetical world where, other things equal, rural children experienced the same mean levels of measured children, primary caregivers, and household exposures as urban children. Our model specification began with a base model of children-level variables (Model 1) and then sequentially added primary caregivers-level covariates (Model 2) and household-level factors (Model 3), to assess the extent to which the rural-urban self-medication in children gaps can be “explained” by these factors. All data was entered into the Epidata Entry software (version 3.1, EpiData Association, Odense, Denmark). Statistical analyses were conducted in STATA version 14. The odds ratio (OR) was reported along with 95% confidence interval (CI). A *p*-value less than 0.05 was considered to be statistically significant.

## Results

### Sample characteristics by rural and urban areas

A summary of the characteristics of 2798 respondents (45.1% in urban, 54.9% in rural) are presented in Table 1. Boys in urban and rural areas accounted for 57.2 and 52.1% (*p* = 0.007). The proportion of children aged 0–2 years and 3–6 years in urban areas was close (50.8 and 49.2%), while the majority of children in rural areas (67.0%) was aged 3–6 years. The majority of children participated in medical insurance (82.1% in urban and 93.0% in rural, *p* < 0.001). In urban and rural areas, 31.7 and 45.2% of children had unhealthy eating habits (*p* < 0.001). There were statistically significant differences between urban and rural areas in children with chronic diseases (5.0 and 2.6%), being sick in the 2 weeks prior to the survey (4.4 and 21.0%) and hospitalized in prior year (5.0 and 9.2%).

**Table 1:** **Characteristics of the study sample: children, primary caregivers, and household in Chengdu of China, stratified, by rural-urban location (*n*** **= 2798)**

Characteristics	Urban areas		Rural areas		
	n	%	n	%	*P* Value
Total	1262	45.1	1536	54.9	
Children					
Gender					0.007
Girls	540	42.8	736	47.9	
Boys	722	57.2	800	52.1	
Age (years)					< 0.001
0–2	641	50.8	507	33.0	
3–6	621	49.2	1029	67.0	
Having medical insurance					< 0.001
No	226	17.9	107	7.0	
Yes	1036	82.1	1429	93.0	
Children having unhealthy eating habits					< 0.001
No	862	68.3	841	54.8	
Yes	400	31.7	695	45.2	
Children with chronic diseases					0.001
No	1199	95.0	1496	97.4	
Yes	63	5.0	40	2.6	
Children being sick in the 2 weeks prior to the survey					< 0.001
No	1207	95.6	1213	79.0	
Yes	55	4.4	323	21.0	
Children hospitalized in prior year					< 0.001
No	1199	95.0	1395	90.8	
Yes	63	5.0	141	9.2	
Primary caregivers					
Gender					0.019
Female	1078	85.4	1358	88.4	
Male	184	14.6	178	11.6	
Age (years)					< 0.001
< =29	199	15.8	314	20.4	
30--44	347	27.5	327	21.3	
45--59	384	30.4	577	37.6	
> =60	332	26.3	318	20.7	
Education					< 0.001
Primary school or below	283	22.4	697	45.4	
Secondary or High school	741	58.7	745	48.5	
College or above	238	18.9	94	6.1	
Occupation					< 0.001
Unemployment	330	26.1	214	13.9	
Nonmanual work	718	56.9	250	16.3	
Manual work	16	1.3	1020	66.4	
Retirement	198	15.7	52	3.4	
Marital status					0.006
Single	129	10.2	112	7.3	
Married	1133	89.8	1424	92.7	
Primary caregivers often acquire health knowledge					< 0.001
No	575	45.6	1015	66.1	
Yes	687	54.4	521	33.9	
Household					
Monthly household income per capita, Yuan					< 0.001
< =1000	90	7.1	1211	78.8	
1001–3000	539	42.7	316	20.6	
> 3000	633	50.2	9	0.6	
Walking time from home to the nearest medical institution					< 0.001
< =10 min	1024	81.1	840	54.7	
> 10 min	238	18.9	696	45.3	

The majority of the primary caregivers were female (85.4% in urban and 88.4% in rural, *p* = 0.019), and married (89.8% in urban and 92.7% in rural, *p* = 0.006). In urban and rural areas, the highest proportion of primary caregivers was 45–59 years old (30.4 and 37.6%, *p* < 0.001). The highest proportion of primary caregivers’ education was secondary school or high school (58.7% in urban and 48.5% in rural, *p* < 0.001). Over half of primary caregivers in the urban areas (56.9%) were engaged in nonmanual work, while more than half (66.4%) of primary caregivers in the rural areas were engaged in manual work. In urban and rural areas, 54.4 and 33.9% of primary caregivers often acquired health knowledge, respectively (*p* < 0.001). The highest proportion of monthly household income per capita was more than 3000 Yuan (50.2%) in urban areas, while the highest proportion of rural areas was less than 1000 Yuan (78.8%). More than half of the respondents took less than 10 min from home to the nearest medical institution (81.1% in urban and 54.7% in rural, *p* < 0.001).

### Prevalence of primary caregivers self-medication for children in urban and rural areas

Table 2 describes primary caregivers self-medication for children in urban and rural areas. A total of 823 (29.4%) primary caregivers self-medicated their children. The results showed that 38.2% primary caregivers in rural areas self-medicated their children, compared to 18.7% of those in urban areas (*P* < 0.001).

**Table 2:** Primary caregivers self-medication for children in urban and rural areas

Self-medication	Urban children		Rural children		***χ***^***2***^	***p***-value
	n	%	n	%		
Yes	236	18.7	587	38.2	127.086	<0.001
No	1026	81.3	949	61.8		

### Factors associated with self-medication in children

Table 3 reports the results of the multivariable logistic regression models predicting the likelihood of self-medication in children, stratified by rural-urban location.

**Table 3:** Multivariable logistic regression analysis of factors associated with primary caregivers self-medication for children in urban and rural areas (*n* = 2798)

Variables	Urban areas		Rural areas	
	OR	95% CI	OR	95% CI
***Children***				
Boys (ref: Girls)	1.15	0.85–1.55	0.99	0.80–1.22
Age (years) (ref: 0–2)	1.36	0.98–1.87	1.51^**^	**1.18–1.94**
Medical insurance (ref: No insurance)				
Social	0.84	0.56–1.26	0.94	0.61–1.46
Commercial	1.36	0.52–3.59	2.56	0.80–8.21
Social and commercial	1.29	0.79–2.11	1.00	0.57–1.75
Children have unhealthy eating habits (ref: No)	2.01^***^	**1.47–2.76**	1.86^***^	**1.49–2.32**
Suffering from chronic diseases (ref: No)	0.64	0.25–1.69	1.99^*^	**1.01–3.89**
Have been hospitalized in the past year (ref: No)	1.40	0.75–2.62	1.06	0.73–1.55
***Primary Caregivers***				
Male (ref: Female)	0.99	0.65–1.51	1.18	0.84–1.66
Age (years) (ref: <=29)				
30--44	0.91	0.58–1.43	1.01	0.72–1.42
45--50	0.74	0.47–1.17	0.75	0.54–1.05
> =60	0.72	0.45–1.14	0.57^**^	**0.38–0.86**
Education level (ref: Primary school or below)				
Secondary and High school	1.39	0.93–2.07	0.82	0.63–1.07
College or above	2.01^*^	**1.17–3.45**	0.42^**^	**0.23–0.78**
Occupation (ref: Unemployment)				
Nonmanual work	0.93	0.64–1.34	0.99	0.65–1.53
Manual work	1.04	0.22–4.93	1.26	0.90–1.76
Retirement	0.66	0.39–1.11	1.32	0.68–2.56
Married (ref: Single)	1.01	0.61–1.67	1.00	0.66–1.51
Primary caregivers often acquire health knowledge (ref: No)	1.07	0.79–1.45	1.04	0.82–1.32
***Household***				
Monthly household income per person (RMB), yuan (ref: <=1000)				
1001–3000	1.04	0.59–1.84	1.42^*^	**1.07–1.88**
> 3000	0.58	0.31–1.06	0.36	0.04–3.00
More than 10 min walk from home to the nearest medical institution (ref: <= 10 min)	1.81^**^	**1.27–2.56**	0.96	0.77–1.19

Notes: * *p* < 0.05; ** *p* < 0.01;***P < 0.001

In urban areas, unhealthy eating habits of children, primary caregivers’ education, and the walking time from home to the nearest medical institution were significantly associated with the self-medication in children. Children having unhealthy eating habits (OR = 2.01, 95% CI: 1.47–2.76) were more likely to have been self-medicated by their primary caregivers. And primary caregivers with college or above education (OR = 2.01, 95%CI: 1.17–3.45), who spent more than 10 min from home to the nearest medical institution (OR = 1.81, 95%CI: 1.27–2.56) were more likely to self-medicate their children.

In rural areas, children’s age, unhealthy eating habits of children, children suffering from chronic diseases, primary caregiver’s age, primary caregiver’s education, and monthly household income per capita were significantly associated with the self-medication in children. Primary caregivers were more likely to self-medicate their children aged 3–6 years (OR = 1.51, 95% CI: 1.18–1.94), having unhealthy eating habits (OR = 1.86, 95% CI: 1.49–2.32), and suffering from chronic diseases (OR = 1.99, 95%CI: 1.01–3.89). Primary caregivers who over the age of 60(OR = 0.57, 95% CI: 0.38–0.86), with college or above education (OR = 0.42, 95% CI: 0.23–0.78) were less likely to self-medicate their children. And primary caregivers with monthly household income per capita of 1001–3000 (OR = 1.42, 95% CI:1.07–1.88) were more likely to self-medicate their children.

### Decomposing rural–urban disparities in self-medication among children

The respective contributions of the children, primary caregivers and household factors to the “explained” part of the self-medication gap in children are showed in Table 4.

**Table 4:** Predicted probability and disparity in self-medication between rural and urban children, and percentage of the difference explained by children, primary caregivers, and household variables

	Rural children vs. Urban children		
	Model 1 Mean((95%CI)	Model 2 Mean((95%CI)	Model 3 Mean((95%CI)
Predicted probability			
Rural children	0.382 (0.358,0.406) ***	0.382 (0.358,0.406) ***	0.382 (0.358,0.406) ***
Urban children	0.187 (0.165,0.209) ***	0.187 (0.165,0.209) ***	0.187 (0.165,0.209) ***
Rural-urban disparity	0.195 (0.163,0.228) ***	0.195 (0.163,0.228) ***	0.195 (0.163,0.228) ***
Percent difference explained	Contribution	Contribution	Contribution
Total difference explained (%)	18.32***	19.86***	32.01**
Children (%)	18.32***	18.48***	18.54***
Gender	−0.17	−0.19	− 0.19
Age (years)	6.25***	6.55***	6.52***
Medical insurance	−0.95	− 0.98	− 0.76
Children having unhealthy eating habits	9.02***	9.16***	9.14***
Children with chronic diseases	−0.22	− 0.29	− 0.32
Children being sick in the 2 weeks prior to the survey	3.66	3.53	3.48
Children hospitalized in prior year	0.73	0.70	0.67
Primary caregivers (%)		1.38	0.46
Gender		−0.20	−0.18
Age (years)		0.87	0.93
Education		0.24	−0.49
Occupation		0.69	0.57
Material status		0.14	0.13
Primary caregivers often acquire health knowledge		−0.36	− 0.50
Household (%)			13.02
Monthly household income per capita, Yuan			9.21
Walking time from home to the nearest medical institution			3.81

Notes: * *p* < 0.05; ** *p* < 0.01;***P < 0.001

The results showed that the base model (Model 1) included only the children characteristics explained 18.32% of rural–urban self-medication gap in children. Once primary caregivers’ variables were included, the explained portion of self-medication gap in children increased to 19.86% (Model 2). Adding the household covariates, the explained portion of self-medication gap in children increased to 32.01%(Model 3). In the full model (Model 3), children variables explained the largest portion of the rural–urban self-medication gap, approximately 18.54%.

The predicted rural–urban difference in probability of self-medication among children was 0.195 (95% CI:0.163,0.228). Children’s age and children having unhealthy eating habits were significant factors in the decomposition model 3. And these two variables contributed positively to the modeled rural–urban gap. Unhealthy eating habits of children were the largest contributor to the part of the inequality deriving from differences in group characteristics. The rural-urban self-medication gap in children would be reduced by 9.14 and 6.52%, respectively, if children in rural areas had the same rate of unhealthy eating habits and age distribution as those in urban areas.

## Discussion

This study is to examine the potential factors associated with self-medication in children. At the same time, this study is the first to explore the potential contribution of children, primary caregivers and household factors to the rural-urban disparities in self-medication among children, and to propose alternative strategies to reduce the rural-urban disparities.

This study found that the rate of primary caregivers self-medication for children in rural areas (38.2%) was higher than that in urban areas (18.7%). These possible reasons may be responsible for the situation in China. One is that prescription medicine management in rural areas is less strict than urban places, and prescriptions might be easier to obtain [27]. The other is that rural residents are usually far away from health facilities, or their families are poor [28], may force them to use leftovers from previous prescriptions or bought at pharmacy to treat their children health disorders [29].

This study found that the education of primary caregivers was the common factor influencing the self-medication among urban and rural children. Urban primary caregivers with college or above education were more likely to self-medicate their children. It was similar to the previous studies found that well-educated primary caregivers preferred to use medicine for their children themselves without seeing doctors [7, 13]. In contrast to urban areas, rural primary caregivers with college or above education were less likely to self-medicate their children. A previous study also indicated that primary caregivers with lower levels of education were more likely to self-medicate their children [30]. This is an interesting paradox that suggests the higher educational level of primary caregivers is a risk factor for self-medication among urban children but a protective factor for self-medication among rural children in China. The influencing mechanism of the primary caregivers’ education on self-medication among children in different regions is worth noting for future research. Unhealthy eating habits of children were also the common enabling factor for primary caregivers self-medication for children in urban and rural areas. Unhealthy eating habits could lead to nutritional imbalance, and thus lead to a decline in children’s health [31]. A previous study found that poor health condition of children was a strong predictor of self-medication in children [32].

In urban areas, walking time from home to the nearest institution was another influential factor which impacted on primary caregivers self-medication for children. In this study, urban primary caregivers who spend more than 10 min from home to the nearest medical institution were more likely to self-medicate their children. Self-medication means lower utilization of medical services. M. Bayham’ study reported that longer travel time to a health facility was associated with decreased utilization of medical services [33].

In rural areas, children’s age, primary caregivers’ age, and the monthly household income per capita were another set of influential factors which impacted on primary caregivers self-medication for children. Primary caregivers were more likely to self-medicate their children who aged 3–6 years. It was similar to a national survey in China, which showed that the proportion of guardians using antibiotics without doctors’ prescription for their children was getting higher with the age of children increases [5]. P. Bi’s study also showed that mothers might become less protective, self-medication and even medicine abuse when the children grow up [13]. The primary caregivers who over the age of 60 years were less likely to self-medicate their children compared with those under the age of 29 years. It was in accordance with some studies identified younger age of primary caregivers to be significantly associated with the increased antibiotic use in self-medication [34–36]. The monthly household income per capita was also a factor influencing the self-medication of rural children. The primary caregivers with monthly household income per capita of 1001–3000 Yuan were more likely to self-medicate their children than those with less than 1000 Yuan. It is possible that they could afford to buy medicine from pharmacies, but have difficulty in affording to visit physicians [37].

In addition, rural primary caregivers were more likely to self-medicate their children suffering from chronic diseases. Hoai et al. also found that chronic disease was positively associated with the frequency of self-medication [38].

R. Li’s study showed that the prevalence of antibiotic use without doctors’ prescription in boys was higher than that in girls in Western and Eastern China, and primary caregivers having basic health knowledge were protective factors of antibiotic in self-medication among children 0–6 years with diarrhea in China [5]. But this study found children’s gender and primary caregivers often acquire health knowledge were not associated with primary caregivers self-medication for children in urban and rural areas.

This study provides new insight into sources of self-medication disparities by utilizing an econometric technique that has been applied to study disparities in other social and economic issues. We found that children, primary caregivers and household variables helped explained 32.01% of the rural–urban self-medication gap in children.

Children variables explained the largest portion of the rural–urban self-medication gap in children. Unhealthy eating habits of children were the largest contributor to the part of the inequality deriving from differences in group characteristics. Characteristics analysis also showed that the rate of unhealthy eating habits in the rural areas was significantly greater than that in the urban areas (45.2%vs.31.7%, *P* < 0.001). And multivariable logistic analysis found that primary caregivers were more likely to self-medicate their children having unhealthy eating habits. Another factor contributing to the children rural-urban self-medication gap was children’s age. The odds of children’s self-medication in aged 3–6 years was greater than those who were aged 0–2 years. Since the children’s self-medication is determined by the primary caregivers, educational programs and public campaigns should be considered by health care authorities in order to ensure the primary caregivers to recognize the dangers of self-medication for children and the importance of a balanced diet to children’s health.

The relative strength and weakness must be taken into account when interpreting the findings of our study. This is the first study to examine the contribution of children, primary caregivers and household factors to the rural-urban differences in self-medication among children, which is conducive to reduce the rural-urban inequity. Our study is also strengthened by our methodology using face-to-face interviews which increases the precision of information obtained. However, it also has some limitations. This study is the cross-sectional study design which does not allow for determination of causality. In addition, this study lacks the survey of factors such as health belief and health literacy of primary caregivers, which may be related to self-medication in children [39].

## Conclusion

This study found that the rate of primary caregivers self-medicated their children in rural areas (38.2%) was higher than that in urban areas (18.7%). Unhealthy eating habits of children were the largest contributor to the rural-urban self-medication inequality in children. The evidence presented in this study increasingly suggests that public health policies addressing rural-urban differences in children’s factors could serve as an effective method for reducing rural-urban disparities in self-medication among children, and therefore, reducing long-term differences in health outcomes later in life. Educational programs and public campaigns should be considered by health care authorities in order to ensure the primary caregivers to recognize the dangers of self-medication for children. As the findings reported, a substantial portion of rural-urban differences in self-medication among children remained unexplained by the uneven distributions of the above factors between rural areas and urban areas. Therefore, future research is strongly recommended to identify and assess the contribution of each unknown factor.

## Data Availability

The datasets generated and/or analysed during the current study are available in the figshare repository, 10.6084/m9.figshare.12416363
